# Efficacy of Liquid Smoke to Mitigate Infestations of the Storage Mite, *Tyrophagus putrescentiae*, in a Model Semi-Moist Pet Food

**DOI:** 10.3390/ani13203188

**Published:** 2023-10-12

**Authors:** Aiswariya Deliephan, Thomas W. Phillips, Bhadriraju Subramanyam, Charles G. Aldrich, Jacqueline Maille, Naomi Manu

**Affiliations:** 1 Department of Grain Science and Industry, Kansas State University, Manhattan, KS 66506, USA; sbhadrir@ksu.edu (B.S.); aldrich4@ksu.edu (C.G.A.); 2 Department of Entomology, Kansas State University, Manhattan, KS 66506, USA; twp1@ksu.edu (T.W.P.); jmaille@ksu.edu (J.M.); nmanu30@ksu.edu (N.M.)

**Keywords:** food safety, liquid smoke, pet food, storage mite, *Tyrophagus putrescentiae*

## Abstract

**Simple Summary:**

Pests such as mites have been contaminating food products, including pet food, over the years. Mite-infested pet food, apart from causing allergies in pet animals and degrading food quality, can also cause illnesses like asthma in humans as they are often handled by pet owners and children at home. In this study, we evaluated liquid smoke as a natural and clean label additive in semi-moist pet food to protect against mite infestation. Our results indicated that while liquid smoke did not kill or inhibit the mite growth, some liquid smoke fractions provided a repellency effect to retard mite infestation in semi-moist pet food.

**Abstract:**

The storage mite *Tyrophagus putrescentiae* infests a wide range of food products including pet food. Control of this mite depends on chemical methods such as fumigation and spraying with insecticides. Methyl bromide was used as a fumigant for high-value stored products, especially to control mite infestation in dry-cured hams and cheeses, but it is now banned for most uses in many countries because of its atmospheric ozone-depleting effects. Effective alternatives to methyl bromide are needed to develop integrated pest management programs for this pest. Liquid smoke is a naturally derived flavoring and preservative with known antimicrobial properties. The objective of this study was to investigate the efficacy of liquid smoke preparations, with varying phenol and carbonyl concentrations and pH, on the survivability and orientation behavior of *T. putrescentiae* in a model semi-moist pet food. The mite survivability assays using liquid smoke-treated and untreated semi-moist pet food samples indicated that there was no difference among treatments (*p* > 0.05) for mite infestation and survival. Two-choice behavioral assays using semi-moist pet food cubes dipped in varying concentrations (0%, 0.3%, 1%, 5%, 10%, 25%, 50%, or 100% *v*/*v*) of liquid smoke preparations found that some of the liquid smoke preparations containing medium to high carbonyl content repelled the mites. In conclusion, liquid smoke did not kill or inhibit the mite population growth in semi-moist pet food. However, some liquid smoke fractions containing medium to high carbonyl content were repellent to mites and may retard mite infestation in stored semi-moist foods.

## 1. Introduction

Some of the most important sources of food allergens worldwide are stored product mites, [1] mainly *Tyrophagus putrescentiae* [2]. This stored food mite is a cosmopolitan species frequently found in a wide variety of stored products. It is commonly known as cheese or mold mite and is also sometimes referred to as ham mite. It is particularly common in foods with high levels of fat and protein, such as dried eggs, ham, fish meal, cheese, nuts, and pet foods [2,3]. It is a significant cause of allergic asthma and allergic rhinitis among grain handlers, bakers, and food industry workers because it occurs in large numbers in farm buildings and in food manufacturing and storage facilities. It is also being recognized as an important contributor to the allergen content in dust in homes [4]. Furthermore, stored food mites are responsible for acute enteritis and severe systemic reactions or anaphylaxis, as a result of the consumption of food that was infested with these mites [5]. Mites are also implicated in the transmission of pathogenic micro-organisms, mold spores, and prions [6,7].

Stored product mites may be considered important allergens in dogs causing atopic dermatitis. In a recent study [8], three species of storage mites including *T. putrescentiae* were captured from the processing and packing areas of a pet food manufacturing factory in the United Kingdom, even in the cleanest locations. This suggests that pet food ingredients or even the finished products could become a suitable source of stored product mites. Commonly, the stored product mites live on the external surface of these food products, but sometimes they penetrate inside, causing serious economic losses [9]. When pet foods are stored at home by the consumers, they are susceptible to mite infestation from house dusts which may harbor some of the storage mites. Brazis et al. [10] observed that two out of ten different brands of sealed commercial dog foods contained storage mites, and upon storing those at an optimal temperature and humidity, nine out of ten of them contained storage mites.

Traditionally, the control of mites depends on chemical methods such as fumigation with methyl bromide, spraying cracks, and crevices with organophosphorus compounds, or treatment with pesticides like benzyl benzoate and repellents like DEET (N,N-diethyl-m-toluamide) [11]. Residual pesticides are mainly used to treat the structure of buildings, and some are applied directly to raw commodities like stored grains. Methyl bromide, though an effective fumigant used to control storage mites like *T. putrescentiae* has been phased out in industrialized nations across the world including the United States due to its ozone-depleting nature [12]. Numerous efficacy tests have been performed with experimental and formulated acaricides against mold mites in the context of agricultural and industrial settings [13,14,15,16,17,18]. They leave behind some amount of residue on the treated commodity, and repeated use of these chemicals has resulted in the development of resistance in mites [19]. These synthetic pesticides have been shown to cause undesirable effects on non-target organisms and have fostered environmental and human health concerns [20]. These problems have highlighted the need to develop new strategies for selective storage mite control, preferably using safe, non-toxic, and natural compounds.

To date, there are no targeted intervention strategies against storage mites that pet food industries could follow beyond the use of propylene glycol as a humectant. However, although propylene glycol added to pet food is very effective in preventing mite infestation, this polyol is not a preferred ingredient by the pet food industry and many consumers as it is poorly tolerated by cats causing Heinz body formation in their blood leading to anemia [21]. Furthermore, some consumers have considered propylene glycol as a controversial additive to use in food products, due to consumers mistakenly assuming it to be the same as the very toxic ethylene glycol, which is a key component in anti-freeze [22]. Replacing propylene glycol with glycerol, which is not toxic to cats, dogs, and people, is a preferred ingredient option. However, *T. putrescentiae* is not affected by glycerol, which then leaves semi-moist pet food with glycerol to be highly susceptible to mite infestation [23]. Therefore, a strategy to use an alternative, such as liquid smoke, might be a new solution.

Liquid smoke is a naturally derived flavor component and preservative used in human and pet foods, with known antimicrobial properties. Liquid smoke is approved by the United States Food and Drug Administration as a “Generally Recognized as Safe” (GRAS) substance and can be included in human and pet foods with no specific limit, and the flavor of liquid smoke is widely likable in these foods as well. There is no available information on its potential as a toxin or repellent against stored product mites. Eischen and Wilson [24] tested wood smoke from 40 different plants on *Varroa* mites, ecto-parasites of honeybees (bee mites), and found two of them to be effective. To our knowledge, there are no studies that tested liquid smoke on the stored product mite *T. putrescentiae.* Ernst et al. [25] reported that coating pet food with conjugated linoleic acid inhibited *T. putrescentiae* growth and infestation. Abbar et al. [23] evaluated *T. putrescentiae* population growth, orientation behavior, egg-laying, and population growth on small dry-cured ham pieces with and without coatings of food-safe additives as alternatives to propylene glycol and found that the GRAS food additives, butylated hydroxytoluene, sodium sorbate, sodium propionate and propionic acid to be very effective. Manu et al. [26] reported that a mixture of three short-chain fatty acids, C_8,_ C_9,_ and C_10_, and the sesquiterpene ketone nootkatone coated on ham cubes repelled *T. putrescentiae*. Other than these, we could not find any published literature that tested the inhibition or repellency effects of GRAS components on *T. putrescentiae* infestation. The objective of this study was to investigate the effects of liquid smoke preparations on the storage mite *T. putrescentiae* population growth and orientation (attraction/aversion) behavior when incorporated into a model semi-moist pet food.

## 2. Materials and Methods

### 2.1. Preparation of Semi-Moist Pet Food and Source of Liquid Smoke

The formula used for the preparation of semi-moist pet food is shown in Table 1. All the ingredients were weighed according to proportion in the formula (Table 1) to produce 2 kg of pet food per batch. A 3.3 L planetary mixer (KitchenAid Portable Appliances, St. Joseph, MI, USA) was used to mix the ingredients at a speed of about 50 rpm for 10 min. The dry ingredients were mixed first, followed by the liquid ingredients. The mixture was spread in a uniform layer approximately 1 cm thick on a baking tray lined with parchment paper. It was baked in a convection oven (MEA 21-93-E; Garland Commercial Industries, Freeland, PA, USA) at 175 °C for 10 min. The baked pet food was cooled on a wire rack until it reached room temperature. It was then cut into uniform cubes of size 3 × 3 × 1 cm using a stainless-steel knife.

Table 2 is a list of eight liquid smoke preparations. These were provided by the sponsor of this study, Kerry Ingredients (Beloit, WI, USA).

### 2.2. Mite Culture

Laboratory cultures of *T. putrescentiae* have been maintained in the Department of Entomology at Kansas State University for more than 4 years and have not been subjected to any pesticides. The mites were reared in glass jars containing a mite diet similar to the culture used in the study by Abbar et al. [23]. The glass jars were sealed with labeled filter paper in the metal lid ring. The mite rearing diet was composed of agar, yeast, cellulose (Alphacel, ICN Biomedicals, Costa Mesa, CA, USA), mixed vitamins (Vanderzant modification vitamin mixture for insect diet; MP Biomedicals LLC, Irvine, CA, USA) (5:5:5:5 g), dog food (Purina Beneful, Nestlé-Purina Pet Care, St. Louis, MO, USA) (160 g), glycerol, antifungal salt solution in ethanol (methyl-p-hydroxybenzoate, 15:85 g mL^−1^) and water (25:25:475 mL), which were mixed and cooked for about half an hour and then added to the dog food in rearing jars. Mites from the already maintained laboratory culture were introduced to new cultures after the diets had been cooled to 25 °C. The jars were stored in an incubator at 25 °C and 70% RH in total darkness.

### 2.3. Treatments Used in the Study

For the mite population growth study (experiment 1), three liquid smoke preparations, P-1720, Cloud S-5, and Cloud S-C100, were evaluated at 0.3% (*w*/*w*) inclusion in the semi-moist pet food. The liquid smoke preparations were chosen based on the supplier’s recommendation that they have been evaluated in pet food as a flavoring. Semi-moist pet food with no added liquid smoke served as the untreated control. A standard mite-rearing diet (not a semi-moist pet food product, and no added liquid smoke), as described above, served as the negative control. Semi-moist pet food dipped in 20% propylene glycol solution for 2 min and air-dried for 1 min served as the positive control. Three replications were performed for this study.

For the mite orientation behavior study (experiment 2), all the liquid smoke preparations, S1, S2, S3, S4, S5, S6, S7, and S8, were evaluated at concentrations of 0.3%, 1%, 5%, 10%, 25%, 50%, or 100% *v*/*v* (by volume in water) as coating on semi-moist pet food for sampling time periods 2, 8, and 24 h, in comparison to untreated control (0% smoke). The coating was performed by dipping the semi-moist food cubes in liquid smoke at the desired concentration levels for 2 min and air-dried for 1 min. A propylene glycol treatment (positive control) was evaluated at 20% concentration as coating on the pet food for 2, 8, and 24 h. Each treatment combination was replicated three times in a completely random design.

### 2.4. Experiment 1: Mite Population Growth Assay

A standard mite diet was prepared using a modified recipe of the mite culture diet described earlier. It was composed of agar, yeast, cellulose, and pre-mixed vitamins at a ratio of 20:5:5:5 g, along with glycerol, antifungal salt solution in ethanol, and water at the ratio of 25:25:475 mL. The ingredients were mixed and cooled to set and cut into 3 × 3 × 1 cm cubes. The standard mite diet was included to determine the reproductive potential of *T*. *putrescentiae*. The untreated and smoke-treated (0.3% of P-1720, Cloud S-5 or Cloud S-C100) semi-moist pet food cubes and the mite diet cubes were placed separately in glass jars (216 mL; Ball Corp., Broomfield, CO, USA) into which 20 adult mites were introduced. For the positive control of a compound known to inhibit mite reproduction, the untreated pet food cubes were dipped in 20% propylene glycol for 2 min, air-dried for 1 min, and then placed in jars. The jars were sealed with labeled filter paper under the jar ring after mite inoculation. To prevent the mites from climbing up to reach the jar ring and filter paper, the top inside rim of each jar was smeared with petroleum jelly. The sealed jars were placed in plastic tubs containing detergent solution to reduce the surface tension of water so that any mites successful at leaving jars were prevented from escaping. The jars were stored in an incubator at 25 °C and 70% RH in total darkness. The number of live mobile mites (mature adults and immature walking mites) in the jars was counted after 7, 14, and 28 days. Each treatment combination was replicated three times.

### 2.5. Experiment 2: Mite Orientation Behavior Assay

**Figure 1:**
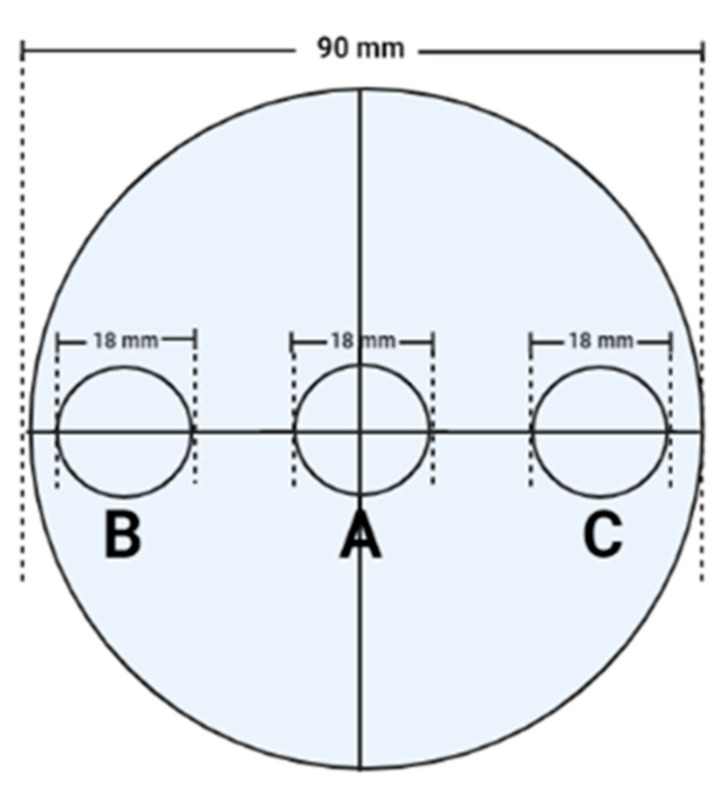
Bioassay arena used in the two-choice behavioral test for mite orientation study. One ‘treated’ pet food cube and one ‘control’ pet food cube were placed in each of the circles, B and C. Twenty adult mites of mixed sex were released into the middle circle A.

Two-choice behavioral assays described by Abbar et al. [23] were conducted using mites to determine whether their orientation towards semi-moist pet food was affected by treatment with liquid smoke. Small cubes of semi-moist pet food (5 × 5 × 5 mm) were used in the two-choice tests. The cubes were placed in bioassay arenas (Figure 1), which consisted of 90 × 20 mm glass Petri dishes that had a 90 mm diameter circular piece of black construction paper covering the floor of each dish on the underside. Three circles of 18 mm diameter were centered along a line passing through the center of the paper, with one circle in the middle of the floor and one circle each at a distance of 5 mm from the side wall. For this study, a ‘treated’ pet food cube was placed in one of the peripheral circles (either A or B) and an untreated ‘control’ pet food cube was placed in the opposite circle at the periphery. Twenty adult mites of mixed sex were released into the middle circle A (Figure 1). The arenas were then placed in total darkness at 25 °C and 70% RH. Mites were prevented from leaving bioassay dishes by applying a thin layer of vacuum grease along the inside upper 5 mm of the vertical wall of the Petri dish bottom. For treated samples, pet food cubes were dipped in 20% propylene glycol or 0.3%, 1%, 5%, 10%, 25%, 50%, or 100% (*v*/*v*) of the liquid smoke preparations S1, S2, S3, S4, S5, S6, S7 or S8, respectively for 2 min. In the control treatment, untreated pet food cubes were dipped in sterile distilled water (0% smoke) for 2 min. The pet food cubes were then air-dried for 1 min before being placed in the bioassay arena. Mites that were oriented towards each of the pet food cubes within the circles or those on the food surface were counted after 2, 8, and 24 h, and these experiments were replicated three times.

The attraction or repulsion of the mites towards smoke treatments was calculated by a repellency index (RI) and expressed as a percentage using the following equation [26,27]:

RI = (N_c_ − N_t_)/T × 100

where N_c_ = number of mites on the untreated control pet food; N_t_ = the number of mites on the liquid smoke-treated pet food; T = total number of mites released (T = 20).

Positive values of RI indicated ‘repulsion’ and negative values of RI indicated ‘attraction’.

### 2.6. Statistical Analysis

For the population growth assay experiment, data were subjected to a two-way analysis of variance (ANOVA), and means among treatments were separated using Tukey’s post hoc test when the *F*-test of the ANOVA per treatment was significant at *p* < 0.05. For the orientation behavioral assay experiments, RI values were subjected to a three-way analysis of variance (ANOVA), followed by two-way ANOVA at each time period. Means among treatments at the 24 h time period were separated using Tukey’s post hoc test when the *F*-test of the ANOVA per treatment was significant at *p* < 0.05. All analyses were conducted using the statistical software SAS version 9.3 [28].

## 3. Results

The mite reproduction assays (Table 3) indicated that at 7 days, the mean mite population on the liquid smoke-treated (P-1720, Cloud S-5, or Cloud S-C100) semi-moist pet food samples ranged from 89 to 95, and that on the untreated pet food, it was was 90 mites on average. The mite culture diet had a mite population of 316 at 7 days. At 14 days, the average mite population among smoke treatments ranged from 207 to 244, which was similar to the untreated (212 mites) semi-moist pet food, but less than that observed on the mite-rearing diet (454 mites). At 28 days, the mite population among the untreated and smoke-treated pet food samples was so large that the number of mites could not be counted accurately. In the propylene glycol treatment (20% propylene glycol; positive control), no mite growth was observed. From the mite population counts in the treatment samples and grouping of treatment means by Tukey’s test (Table 3; *F* = 64.91; df = 5, 24; *p* < 0.05), it was concluded that the liquid smoke preparations tested did not kill mites, did not significantly inhibit their reproduction, and as a result, did not limit mite population growth.

For the two-choice behavioral assays (Table 4), at 5%, 10%, 50%, and 100% smoke concentration, the mean mite repellency index was the highest (20–60% RI) for Cloud S-C100 and Cloud S-AC15. At 1% and 0.3% smoke concentration, the repellency index was the highest (10–20% RI) for Cloud S-5 and Code V among all time periods. At 25%, 50%, and 100% smoke concentration, P-1720, Hickory OS-1473, and Code 10 attracted mites the most (−30 to −40% RI). At 10% smoke concentration, Cloud S-5, Black deli, and Code V attracted mites the most (−50% RI) among all time periods. At 5% and 1%, Hickory OS-1473 and Black deli attracted mites the most (−40 to −50% RI), respectively, whereas at 0.3%, Code 10 attracted mites the most with an RI of −40%. From Table 4, grouping of treatment means by Tukey’s test at 24 h time period (*F* = 4.15; df = 14, 41; *p* < 0.05) indicated that, across different concentrations of liquid smoke treatments, Cloud S-C100 and Cloud S-AC15 containing medium to high carbonyl content were the most effective at repelling the mites followed by Cloud S-5 (low carbonyl, no phenol) and Code V (medium carbonyl, low phenol) smoke preparations. The least effective treatments to repel mites were P-1720, Black deli, Hickory OS-1473, and Code 10.

## 4. Discussion

The storage mite *T. putrescentiae* readily infests foods with a high fat or protein content and moisture content in the range of 15 to 40% [29]. Therefore, semi-moist pet food is a highly suitable model food substrate for investigating mite-inhibiting or no-preference properties of food preservatives and additives like liquid smoke. The results of this study provide information regarding the relationship between the effects of various liquid smoke treatments on mite population growth and their attraction or aversion behavior towards the liquid smoke preparations.

The results from the population growth experiments demonstrated that the three liquid smoke preparations tested in semi-moist pet food at 0.3% inclusion *w*/*w*, namely Cloud S-5, P-1720, and Cloud S-C100, did not reduce or inhibit the population growth of mites when compared to the untreated pet food. These liquid smoke preparations had carbonyl content ranging from low and medium to high. These were incorporated in the semi-moist pet food at 0.3% by weight, as it was the inclusion level recommended by the supplier based on acceptability by pets (dogs) and feasibility. The number of mites on the smoke-treated and untreated pet food samples by day 7 was in the range of 80 to 100 from an initial inoculation of 20 mites, and by day 14 the mite counts were in the range of 200 to 300. By day 28, the mites were innumerable to count. The population doubling time for *T. putrescentiae* under optimum growth conditions of 30 °C and 90% RH was about 1.75 days [30]. Accordingly, mite growth in our study at 25 °C and 70% RH on the mite-culturing diet was about 300 mites on day 7 and 450 mites on day 14, which was a little lower, possibly due to the lower temperature and relative humidity conditions. However, the mite-rearing diet had a higher moisture content of 70% compared to the semi-moist pet food, which had a 26% moisture content. This might explain the slightly lower mite population growth on the semi-moist pet food samples compared to the mite diet, even though the temperature and relative humidity conditions were the same for both. Our mite count results were similar to the results obtained by Abbar et al. [23], who tested mite population growth on dry-cured ham pieces with and without coating by various food-safe components. Their control treatment, which was untreated dry-cured ham pieces coated with water, had similar counts (200–300 mites) at the end of two weeks. We believe that the 0.3% inclusion level of smoke in semi-moist pet food could be too low of a concentration level to cause any inhibitory effect on mite population growth. Unfortunately, incorporating very high concentrations of smoke in pet food is impractical and not feasible due to the strong flavor and aroma that could be unpalatable for pets.

As it was determined from the population growth assay that three of these liquid smoke treatments at an inclusion level of 0.3% in semi-moist pet food did not inhibit mite population growth compared to the diet with propylene glycol, the next experiment was designed to determine the attraction or repellency effect of the various liquid smoke preparations at different concentration levels on a wider range (0.3% to 100%) using two-choice behavioral assays. For the behavioral assays, the smoke treatments S1 to S8 were tested as coatings on the semi-moist pet food across a wide range of concentrations (0.3%, 1%, 5%, 10%, 25%, 50%, and 100% *v*/*v* of water). The exterior coating of smoke treatments on semi-moist pet food pieces, or on the inside surfaces of commercial packages, can also be a feasible approach to apply higher concentrations and test their repellency effects on mites. Our work here found that it was a surface application of the smoke additive, which was not incorporated within the food, that kept mites away from those food pieces. Surface application to process foods after cooking, baking, or heat extrusion means less possible degradation of the smoke components by the other ingredients in the pet food recipe, which at the same time can also have a higher concentration effect as it is applied only to the surface of the pet food sample.

Measuring the activity of a repellent on insects and mites using behavioral tests has been reported by several researchers [31,32,33,34,35,36]. These researchers studied the repellency effects of volatile organic compounds and essential oils of plant origin on different species of mites using different types of choice-behavioral test strategies, namely Y-tubes [37], T-tubes [35], four-way olfactometers [38,39], two pieces of repellent-impregnated filter paper in a Petri dish [34], or cardboard traps impregnated with the repellent chemical [31]. To our knowledge, mite repellency effects of liquid smoke preparations have been investigated for the first time in this study. We used two-choice behavioral assays similar to the study by Abbar et al. [23] using the liquid smoke-coated food sample on one side and the control food sample on the other side in a glass Petri dish (bio-assay arena). The results from our repellency studies showed that mites avoided semi-moist pet food samples coated with two or three liquid smoke coatings tested at concentrations of 5%, 10%, 50%, and 100%. These smoke preparations had medium to high carbonyl content, some organic acid, and low phenol content. Cloud S-5 (low carbonyl, no phenol) and Code V (medium carbonyl, low phenol) smoke preparations also repelled mites at low concentrations (1% and 0.3%). It was surprising that these two liquid smokes did not repel mites at some of the higher concentrations (10% and 25%) tested. The reason could be that the mites were introduced in the middle region of the bioassay arena, with the control and smoke-treated pet food samples on either side. We speculate that when suddenly confronted with the volatile, attraction/repulsion-inducing compounds present in liquid smoke, the mites started moving away from both the control and smoke-treated samples and wandered off outside the test region (the three circles A, B, and C of 18 mm diameter) of the arena. This is a disadvantage of the two-choice bio-assay arena because it provided enough empty space for the mites to wander away from the test area where the food samples were kept. To overcome this disadvantage to some extent, we enumerated the mite counts on and in the circles of the control and smoke-treated samples at three different time intervals: 2 h, 8 h, and 24 h. This would give the mites a longer time (24 h) to become accustomed to the volatile compounds in the arena, make a choice, and move towards their target food sample. The smoke preparations P-1720, Black deli, Hickory OS-1473, and Code 10 were the least effective at repelling the mites with negative repellency indices. It was also surprising that some liquid smokes like Cloud S-5 and Code V had the highest repellency indices at some of the concentration levels (1% and 0.3%), but also attracted mites at another concentration level (10%). These results suggest that the intensity of repellency (or attraction) can depend on the number and physiological status of mites used in the behavioral assays, the concentration and ratio of various compounds present in the liquid smoke preparation, and the temperature and humidity conditions [36,39,40]. Visser [40] reported that starved and satiated phytophagous insects did not behave similarly in terms of seeking host plants. We believe that the satiety of mites we used from the laboratory culture can influence their movement towards or away from the test food samples used in our behavioral study. Also, we introduced 20 adult mites in the assays, so the repellency indices we obtained were based on this limited number of mites’ movement in a span of 24 h. Deletre et al. [39] suggest that the effect induced by a compound depends on its concentration and the duration of exposure to mites. Lee et al. [36] studied the repellency effects of cinnamon oil, clove oil, and their volatile organic compounds depending on the evaporation time against poultry red mites. They showed that both the essential oils were repellent regardless of evaporation time; however, two components of clove oil, namely eugenol and eugenol acetate, were found to change from having a repellent to an attractant effect over time in the experiment. They proposed that this may be partly explained by a difference in the dynamics of evaporation between the single compounds and the whole essential oil. Liquid smoke also contains several components from wood/plants like phenols, carbonyls, and organic acids along with volatile compounds that may have different evaporation dynamics, thereby changing their effects over time. Despite these, the smoke preparations Cloud S-C100 and Cloud S-AC15, followed by Cloud S-5 and Code V, had quite high repellency indices (up to 60% RI) at most of the concentrations tested, which enabled their use as repellents to some extent in semi-moist foods.

Plants have been used for centuries in the form of crude fumigants where they are burnt to drive away mosquitoes. They have repelling constituents like alkaloids, terpenoids, and phenolics that target predators that attack them, and wood smoke is known to contain these compounds as well. Baltes et al. [41] found the major proportion of commercial full-strength liquid smoke to be composed of water (11–92%), tar (1–17%), acids (2.8–9.5%), carbonyl-containing compounds (2.6–4.6%), and phenol derivatives (0.2–2.9%). However, in the manufacturing of liquid smoke, a variety of ingredients may be used, such as salts, fatty acids, fatty esters, and carriers like saccharides [41,42]. Phenolic compounds contribute to the smoke flavor and color of liquid smoke, and they also have antibacterial and antioxidant properties [43,44,45]. Carbonyl-containing compounds impart a sweet or burnt-sweet aroma and tend to soften the heavy smoky aroma associated with phenolic compounds with some ‘typical smoke-cured’ aromas and flavors [46,47,48]. Wood smoke, phenolic compounds in plant essential oils, and fatty acids were found to repel or inhibit the growth of mites by several researchers. Eischen and Vergara [49] tested the efficacy of smoke from various plant materials, including gobernadora, eucalyptus, coffee beans, corncobs, pine needles, and tobacco on *Varroa* mites (bee mites), and found that smoke from creosote bush caused 60% to 100% mortality and that from grapefruit leaves repelled mites. Masoumi et al. [50] studied the repellent effects of carvacrol and thymol, the essential oils found in oregano and thyme, against mites. Carvacrol is a monoterpenoid phenol and thymol is a monoterpene, both known for their acaricidal properties [51,52,53,54]. Ernst et al. [25] reported the inhibition of *T. putrescentiae* growth in pet food when treated with conjugated linoleic acid. Smoke is also known to contain terpenoids, phenolics, and fatty acids. We hypothesize that the ratio and composition of carbonyls to phenols to volatile organic compounds in liquid smoke might be responsible for the repellency effect on mites in our behavioral assays.

The mechanism of attraction or repulsion towards chemicals by mites has been described by Carr and Roe [55]. Mites use chemosensory sensilla present on their palps and tarsi to identify olfactory and gustatory chemicals in the environment. Olfactory sensilla detects volatilized chemicals, whereas gustatory sensilla detects chemicals via direct contact [56]. It is hypothesized that the molecular mechanism involved in the binding of chemical molecules and neuron depolarization in olfactory and gustatory receptor cells share a similar signal cascade mechanism, though the exact proteins involved are still unknown [55]. We propose the repellency effect on the mites from the phenolic and other volatile compounds present in liquid smoke happens according to the two-step mechanism described by Deletre et al. [39] for chemical/attractant response. The first step is the “choice” of host or favorable food substrate and consists of searching for and recognizing the food by means of olfactory and/or visual clues/stimuli. This choice is made at a distance from the food source. The second step is the ‘selection’ of the host/food, which consists of contacting and accepting the food and, in some cases, selecting a suitable feeding area on the food surface based on contact chemoreception (or ‘taste’). The repellency or attraction takes place through stimuli of receptors in the mites’ body and can be influenced by the chemical/volatile compounds present in liquid smoke.

*T. putrescentiae* is also called mold mite as the presence of mold on food substrates helps proliferate this mite species as it feeds on the mold. In previous works by Deliephan et al. [57,58,59], it was reported that liquid smoke can be used in pet food to enhance food safety, and the liquid smoke preparations Cloud S-C100 and Cloud S-AC15 containing medium to high phenols had a fungistatic and fungicidal effect on the growth of the storage mold *Aspergillus flavus* in semi-moist pet food at concentration levels of 1% to 2%. This mold-inhibiting potential of liquid smoke can be an added advantage to deter mite infestation in semi-moist foods with the inclusion of liquid smoke. For future work, the appropriate concentration levels of inclusion of liquid smoke in semi-moist pet food need to be determined using palatability tests with pet animals for practical application. A modified two-choice behavioral assay using T-tubes can be conducted, which is more suitable for mite repellency tests, and an in-depth analysis of individual components in liquid smoke that can cause repellent effect also needs to be investigated to better understand the effects of liquid smoke on mites. The characterization of the components of liquid smoke would help in exploring the use of combinations of smoke preparations to study their antagonistic and synergistic effects against target pests. The method of application of liquid smoke could also influence its efficacy. For example, a recent study by Shao et al. [60] reported that liquid smoke when applied along with xanthan gum either as coating or in the netting on dry-cured ham was effective in controlling mite infestation. However, without xanthan gum, liquid smoke was not effective against the mites when applied as coating even at higher concentrations. When applied in the netting, it was effective without xanthan gum. For future work, the different ways of applying liquid smoke to pet foods and their synergistic effects with other food ingredients can also be investigated.

## 5. Conclusions

Methyl bromide has been an effective fumigant to control *T. putrescentiae* and other storage mite infestation in foods [61]. However, it has been banned in several countries including the United States due to its ozone-depleting nature. DEET is also one of the most used active ingredients in insect and mite repellents [62]. As the use of such insecticides and fumigants is less safe in spaces like retail food stores and consumers’ homes where food is stored, it is more practical and safer to use food-safe components like liquid smoke in semi-moist pet foods. In this study, liquid smoke did not kill or inhibit the mite population growth; therefore, it will not replace chemical fumigants at this time. However, the liquid smoke preparations Cloud S-C100 and Cloud S-AC15, which contain medium to high carbonyl concentration and low phenols, may provide some repellency effect and retard mite infestation in stored semi-moist pet food when evaluated as coatings on semi-moist pet food. We propose that the use of these liquid smoke preparations in semi-moist foods along with good manufacturing practices (GMPs) and sanitation practices by the food industries would help in developing an effective integrated pest management program to control storage mite infestation.

## Figures and Tables

**Table 1:** Formula used for manufacturing the model semi-moist pet food.

Ingredient	Percent *w*/*w*
Water	18.0
Corn	12.2
Chicken by-product meal	11.4
Corn gluten meal	11.4
Glycerin	12.2
Wheat	5.8
Choice white grease	5.8
Corn syrup	5.0
Gelatin	5.0
Rice flour	4.3
Soybean meal	4.3
Molasses	1.0
Dry dog digest	0.5
Salt	0.5
Dicalcium phosphate	1.4
Vitamin premix	0.2
Phosphoric acid	0.2
Potassium chloride	0.2
Lysine	0.1
Trace mineral premix	0.1
Calcium carbonate	0.1
Choline	0.1
Potassium sorbate	0.1
Natural antioxidant	0.1
Total	100.0

**Table 2:** Liquid smoke preparations evaluated in the study.

Liquid Smoke Preparation	Name	Description
S1	P-1720	Buffered low phenol smoke, medium carbonyl
S2	Cloud S-5	Buffered pH, low acid, low carbonyl, no phenol
S3	Cloud S-C100	Carbonyl preparation: high carbonyl, low acid, very low phenol
S4	Black deli	Basic pH smoke, zero carbonyls, organic acid salts, phenols
S5	Hickory OS-1473	Phenol preparation: high phenol, low acid, no carbonyl
S6	Code 10	Base smoke: organic acid/carbonyls/phenols
S7	Code V	Organic acid preparation: low pH, medium acid, medium carbonyl, low phenol
S8	Cloud S-AC15	High-buffered organic acid + medium carbonyl preparation

**Table 3:** Mean mite population growth at 7 and 14 days on semi-moist pet food treatments with the inclusion of liquid smoke preparations, namely P-1720, Cloud S-5, and Cloud S-C100 at 0.3% *w*/*w*, in comparison to the untreated control (semi-moist pet food with no smoke added), and the mite rearing diet (lab culture diet). Mite population at 28 days was innumerable to count (not shown). Propylene glycol treatment (positive control) did not have mite population growth.

Treatment	Mite Population (Mean ± SE) ^1,2,3^	
	Day 7	Day 14
Mite diet	316.7 ± 32.1 ^a,A^	454.0 ± 50.5 ^b,A^
Untreated	90.3 ± 27.2 ^a,B^	212.0 ± 59.9 ^b,B^
P-1720	95.0 ± 30.4 ^a,B^	244.0 ± 32.4 ^b,B^
Cloud S-5	89.3 ± 31.9 ^a,B^	207.7 ± 34.2 ^b,B^
Cloud S-C100	91.7 ± 23.2 ^a,B^	216.7 ± 32.0 ^b,B^

^1^ Each mean is based on *n* = 3 replications. ^2^ Means among the treatments across day 7 and day 14 followed by different letters in lower case are significantly different (*p <* 0.05, Tukey’s test). ^3^ Means among treatments within each time period of 7 or 14 days followed by different letters in upper case are significantly different (*p <* 0.05, Tukey’s test).

**Table 4:** Mean mite repellency indices for the liquid smoke preparations at 100%, 50%, 25%, 10%, 5%, 1%, and 0.3%, coated on semi-moist pet food at enumeration time points 1 h, 8 h, and 24 h. Semi-moist pet food coated with distilled water served as the untreated control and 20% propylene glycol treatment served as the positive control. Positive control showed 100% repellency. RI values which are “+” indicate repellency and “−” indicate attraction.

Treatment	Time (h)	Repellency Index (Mean ± SE) ^1,2,3^						
		Concentration						
		0.3%	1%	5%	10%	25%	50%	100%
P-1720	2	−25 ± 5	10 ± 4	−20 ± 3	20 ± 6	−20 ± 3	−50 ± 4	−40 ± 5
	8	−30 ± 3	20 ± 6	−20 ± 3	0 ± 4	−20 ± 5	−40 ± 4	−40 ± 3
	24	−40 ± 3 ^a,B^	0 ± 4 ^a,B^	−30 ± 5 ^a,B^	−10 ± 3 ^a,B^	−30 ± 6 ^a,B^	−40 ± 3 ^a,B^	−40 ± 4 ^a,B^
Cloud S-5	2	10 ± 6	0 ± 3	−10 ± 4	−20 ± 5	0 ± 4	20 ± 6	10 ± 3
	8	10 ± 4	10 ± 5	0 ± 4	−40 ± 3	−20 ± 6	20 ± 4	0 ± 5
	24	20 ± 3 ^a,A,B^	20 ± 6 ^a,A,B^	0 ± 3 ^a,A,B^	−50 ± 3 ^a,A,B^	−20 ± 4 ^a,A,B^	10 ± 3 ^a,A,B^	0 ± 6 ^a,A,B^
Cloud S-C100	2	−10 ± 5	10 ± 4	20 ± 6	30 ± 6	10 ± 3	50 ± 4	50 ± 5
	8	0 ± 3	10 ± 6	30 ± 4	30 ± 4	−10 ± 5	60 ± 4	50 ± 3
	24	10 ± 3 ^a,A^	10 ± 4 ^a,A^	25 ± 3 ^a,A^	20 ± 3 ^a,A^	−10 ± 6 ^a,A^	60 ± 3 ^a,A^	40 ± 3 ^a,A^
Black deli	2	10 ± 3	0 ± 6	−10 ± 3	−20 ± 3	10 ± 4	−20 ± 3	0 ± 6
	8	−10 ± 4	10 ± 5	40 ± 4	−20 ± 3	−20 ± 6	0 ± 4	10 ± 5
	24	−20 ± 6 ^a,B^	−50 ± 3 ^a,B^	10 ± 4 ^a,B^	−50 ± 5 ^a,B^	0 ± 4 ^a,B^	−20 ± 6 ^a,B^	0 ± 3 ^a,B^
Hickory OS-1473	2	−20 ± 5	20 ± 4	−50 ± 3	−20 ± 6	−40 ± 3	−20 ± 4	10 ± 5
	8	0 ± 3	−40 ± 4	−20 ± 5	−30 ± 3	−40 ± 6	20 ± 3	−20 ± 4
	24	0 ± 3 ^a,B^	−10 ± 6 ^a,B^	−40 ± 3 ^a,B^	−30 ± 4 ^a,B^	−40 ± 5 ^a,B^	−30 ± 4 ^a,B^	−40 ± 3 ^a,B^
Code-10	2	−25 ± 5	10 ± 4	−20 ± 3	20 ± 6	−20 ± 3	−50 ± 4	−40 ± 5
	8	−30 ± 3	20 ± 6	−20 ± 3	0 ± 4	−20 ± 5	−40 ± 4	−40 ± 3
	24	−40 ± 3 ^a,B^	0 ± 4 ^a,B^	−30 ± 5 ^a,B^	−10 ± 3 ^a,B^	−30 ± 6 ^a,B^	−40 ± 3 ^a,B^	−40 ± 4 ^a,B^
Code-V	2	10 ± 6	0 ± 3	−10 ± 4	−20 ± 5	0 ± 4	20 ± 6	10 ± 3
	8	10 ± 4	10 ± 5	0 ± 4	−40 ± 3	−20 ± 6	20 ± 4	0 ± 5
	24	20 ± 3 ^a,A,B^	20 ± 6 ^a,A,B^	0 ± 3 ^a,A,B^	−50 ± 3 ^a,A,B^	−20 ± 4 ^a,A,B^	10 ± 3 ^a,A,B^	0 ± 6 ^a,A,B^
Cloud S-AC15	2	−10 ± 5	10 ± 4	20 ± 6	30 ± 6	10 ± 3	50 ± 4	50 ± 5
	8	0 ± 3	10 ± 6	30 ± 4	30 ± 4	−10 ± 5	60 ± 4	50 ± 3
	24	10 ± 3 ^a,A^	10 ± 4 ^a,A^	25 ± 3 ^a,A^	20 ± 3 ^a,A^	−10 ± 6 ^a,A^	60 ± 3 ^a,A^	40 ± 3 ^a,A^

^1^ Each mean is based on *n* = 3 replications. ^2^ Means among treatments across different concentrations at 24 h followed by different letters in lower case are significantly different (*p <* 0.05, Tukey’s test). ^3^ Means among treatments at 24 h at each concentration followed by different letters in upper case are significantly different (*p <* 0.05, Tukey’s test).

## Data Availability

Data will be provided upon reasonable request by author Aiswariya Deliephan.
